# New Trends in Emotion Recognition Using Image Analysis by Neural Networks, A Systematic Review

**DOI:** 10.3390/s23167092

**Published:** 2023-08-10

**Authors:** Andrada-Livia Cîrneanu, Dan Popescu, Dragoș Iordache

**Affiliations:** 1 Faculty of Automatic Control and Computers, University Politehnica of Bucharest, 060042 Bucharest, Romania; andrada.cirneanu@mta.ro; 2 The National Institute for Research & Development in Informatics-ICI Bucharest, 011455 Bucharest, Romania; dragos.iordache@ici.ro

**Keywords:** facial emotion recognition, neural network, deep learning, artificial intelligence

## Abstract

Facial emotion recognition (FER) is a computer vision process aimed at detecting and classifying human emotional expressions. FER systems are currently used in a vast range of applications from areas such as education, healthcare, or public safety; therefore, detection and recognition accuracies are very important. Similar to any computer vision task based on image analyses, FER solutions are also suitable for integration with artificial intelligence solutions represented by different neural network varieties, especially deep neural networks that have shown great potential in the last years due to their feature extraction capabilities and computational efficiency over large datasets. In this context, this paper reviews the latest developments in the FER area, with a focus on recent neural network models that implement specific facial image analysis algorithms to detect and recognize facial emotions. This paper’s scope is to present from historical and conceptual perspectives the evolution of the neural network architectures that proved significant results in the FER area. This paper endorses convolutional neural network (CNN)-based architectures against other neural network architectures, such as recurrent neural networks or generative adversarial networks, highlighting the key elements and performance of each architecture, and the advantages and limitations of the proposed models in the analyzed papers. Additionally, this paper presents the available datasets that are currently used for emotion recognition from facial expressions and micro-expressions. The usage of FER systems is also highlighted in various domains such as healthcare, education, security, or social IoT. Finally, open issues and future possible developments in the FER area are identified.

## 1. Introduction

Over the past years, the automatic process of facial emotion recognition (FER) has become a substantial area of interest for researchers. The main goals for FER systems are the identification of a person’s emotions and their intensities, followed by the classification of expression cause, which can be genuine or simulated.

From the implementation perspective, in the last years, FER systems developed using different types of artificial neural networks (ANNs), which proved to have better results than using traditional machine learning methods based on feature descriptors such as histogram of oriented gradients (HOG), or local binary pattern (LBP) combined with data classifiers such as support vector machine (SVM), k-nearest neighbors (KNN) or random forest. As demonstrated in other detection or recognition processes based on ANNs, people’s emotions can also be accurately detected and recognized in a subject-independent way by building a model through the analysis of a collection of training data from different individuals, including skeletal movements [1]. The use of ANNs for emotion detection and recognition opened many opportunities for practical applications, especially in fields such as healthcare, security, business, education, or manufacturing.

According to Ekman and Friesen [2], there are six fundamental emotions that are easy to recognize: anger, fear, sadness, happiness, surprise, and disgust. On the other hand, what is difficult to label is their veracity and their voluntary control (whether they are simulated or not), which can generate confusion in the identification process of these basic emotions. Further, starting from the basic emotions, derived emotions can be obtained either by varying the intensity degree of the basic emotions (for example, fear can become fright, happiness can become pleasure, etc.) or by combining the basic emotions (for example, surprise and happiness become pleasant surprise). Ekman and Friesen’s model proposes the idea that the generation and interpretation of certain facial expressions are deeply inscribed in the brain and universally recognized. Therefore, these facial expressions are not cultural elements, specific to a nation.

To identify an emotion, the Facial Actions Coding system proposed by Ekman and Friesen [3] describes a set of 46 Action Units (AU) that correspond to the elementary movement of facial muscles. These action units are linked to one muscle, a set of muscles, or a complex movement, and the movements of a certain muscle determine the activation of a certain action unit. Consequently, single or several action units participate in the formation of a facial expression, and the seven emotions are represented by different sets of valid action units.

Further, a systematic review of the scientific studies on emotion recognition from facial expressions, led by psychologist and neuroscientist Lisa Feldman Barrett [4], found that there is no reliable way in which a person’s emotional state can be accurately predicted. However, all proposed emotion recognition systems are based on a similar set of features and well-founded assumptions; there are a small number of distinct and universal emotional categories, the emotions are involuntarily revealed on people’s faces, and they can be detected by algorithms.

Generally, the facial analysis process for emotion recognition is based on the identification, in the analyzed images, of features that represent a set of regions of interest, and which hold important information for a specific emotion [5]. By analyzing the emotion’s formation dynamic over time in multiple images, the features can be classified as temporary (location around the eyes, eyebrows, mouth, cheekbones) or permanent (hair, skin texture) [6]. Moreover, the geometric deformation of these features indicates the emotion intensity level. In the end, emotions are mostly revealed by the deformation of temporary features, but there are also some significant challenges such as head position variations, lighting variations, alignment errors, or occlusions that can affect the recognition process [7].

Facial analysis based on neural networks can vary from full-face processing and analysis to specific facial landmark processing [8]. The full-face analysis approach involves having many different images of the person’s face, whereas in the facial landmark-based approach, the neural networks are trained on facial landmarks such as the right eye, left eye, etc., and the recognition is based on the geometric relationship between the landmarks [9]. 

**Figure 1:**
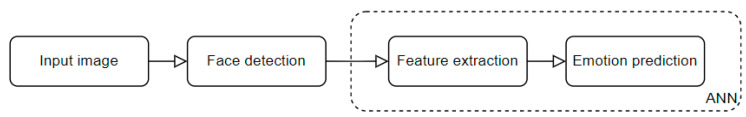
Main components of a facial emotions recognition system based on ANN.

The standard process for emotion detection and recognition from an input image based on ANNs is composed of the face detection component followed by the feature extraction and emotion prediction sub-components of the integrated ANN (Figure 1).

Firstly, face detection can be implemented in several ways:

- a holistic approach—the face is modeled as a whole, without component parts that could be isolated [10];
- component-based approach—certain face attributes can be processed individually [11];
- the configuration-based approach—the spatial relationships between the components of the face are modeled, for example, left eye–right eye, nose–mouth [12].

After the face detection phase, the feature extraction phase performed by different types of learning methods (supervised/unsupervised/reinforcement) proved its usefulness by the fact that in this case, the features are chosen automatically by learning and the performance obtained is superior to traditional methods such as principal component analysis, local feature analysis, or linear discriminant analysis [13,14]. However, some less pleasant aspects are also worth mentioning, for example, the need for many examples to avoid overfitting and the choice of architecture, which can be problematic due to its complexity. Further, the features are determined either on the entire facial area or on specific areas of interest, which can generate problems such as insufficient labeled training data or a challenging labeling process caused by complex or ambiguous training data [15,16]. Nevertheless, in the facial analysis domain, these issues can be overcome using pre-trained networks, semi-supervised learning, or synthesizing new images [17]. Finally, ANN is used to extract significant and non-redundant features and to execute the emotion recognition task, followed by the labeling of the detected emotion with the predicted value.

Nowadays, a powerful form of machine learning is deep learning technology, and it represents a very important aspect in the development of any system that has the requirement to classify specific data such as text or images [18,19]. The success of this technology is generated primarily by the availability of a huge amount of data combined with the technological evolution in terms of data storage and capacity management [20,21]. From the architectural point of view, deep learning is represented by an artificial neural network with many hidden layers between input and output, and it consists of a complex collection of functions that link the layers. In computer vision, the simplest example is the classification of an image to a specific class, which means the network is built on top of a function or multiple functions that have the purpose of mapping the image data to a specific class.

Deep neural networks (DNNs) are the most used machine learning solution by FER systems [22]. DNN uses a system of layers of neurons whose weights are dynamic and changing to match incoming information. Deep learning techniques are used in many FER applications due to the results obtained, results that in some cases exceed the results of the best human subjects. The major advantage of DNN over traditional machine learning techniques is the fact that DNN incorporates the feature extraction step of the input elements, whereas this step is usually performed separately by a domain expert in traditional machine learning techniques [23]. 

This paper is a comprehensive survey of neural network solutions for emotion recognition. In this context, it aims to provide a guide by reviewing the recent developments of FER systems based on neural networks and to provide insights on how to make improvements in this fast-growing field.

The rest of this article is organized as follows. Section 2 presents the methodology for selecting the articles that are included in this survey. An overview of the databases used in neural network-based FER systems is presented in Section 3. Several types of different neural network architectures used in FER systems and the new trends in using neural networks for emotion recognition are presented and discussed in Section 4. A detailed presentation of the use of the FER system is presented in Section 5. Moreover, some challenges, opportunities, and a summary of the advantages and limitations of the FER systems are discussed in Section 6. Section 7 presents the conclusions. A list of abbreviations is provided in abbreviations part.

## 2. Methodology

This review focuses on the latest neural network-based solutions developed for the recognition of specific facial emotions. In this sense, SCOPUS and Web of Science databases were used to identify relevant papers, and then, the results were conducted and reported with reference to the Preferred Reporting Items for Systematic Reviews and Meta-Analyses extension for Scoping Reviews (PRISMA-ScR) [24].

**Figure 2:**
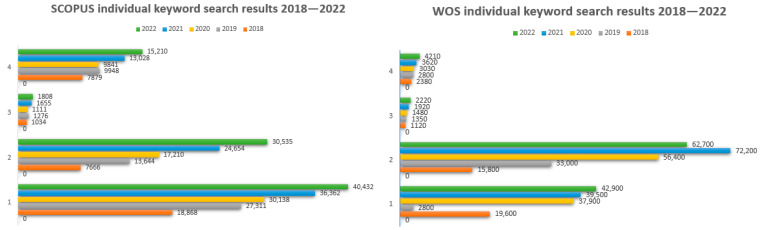
SCOPUS and Web of Science search results on keywords between 2018 and 2022: neural networks, deep learning, emotion recognition, images classification, separately.

**Figure 3:**
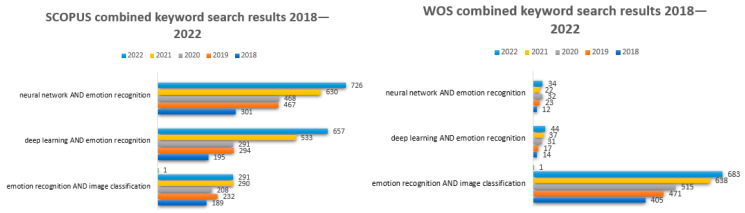
SCOPUS and Web of Science search results on combined keywords between 2018 and 2022.

The search was split between individual keywords (Figure 2 and Figure 3), such as 1—“neural networks”, 2—“deep learning”, 3—“emotion recognition”, 4—“images classification”, as well as combinations of keywords using the “and” connector while searching the title, abstract, and keywords of those original articles. *The resulting collection of articles was filtered based on the publishing year (within the* 2018–2022 period) and the used language (English). After this, duplicates were removed, titles and abstracts were screened and, in the end, the full content of each article was reviewed.

**Figure 4:**
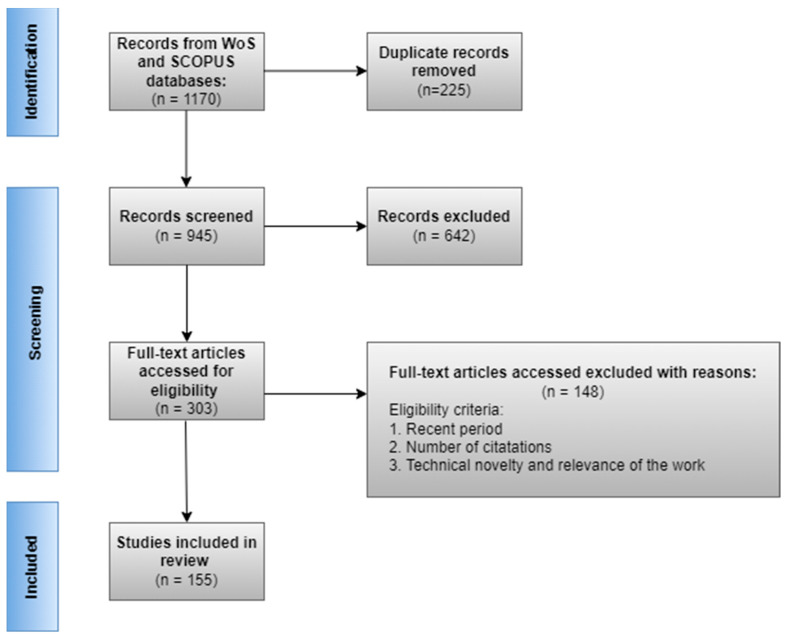
PRISMA flow diagram of the research.

After an initial set of 1170 articles, 945 were screened after the removal of duplicates. Then, 642 articles were excluded after screening titles and abstracts, and 303 articles were excluded after a full content review. The final set is represented by 155 articles. The papers were grouped according to the main and secondary topics addressed: neural network architecture, number of recognized emotions, application field, used databases, and the presented limitations of the proposed methods. The flow of information through the scoping review is presented in Figure 4.

The relevant papers were the ones published in high-ranking conferences and journals and with a considerable number of citations, even though taking into account the number of citations meant filtering out recent papers that did not accumulate citations because of the time constraint. After that, the technical novelty and relevance of the work were the next criteria. Since the survey structure includes sections that can be found in the articles selected for analysis, we believed that the articles’ presentation should be included in the tables for an easier understanding of the solutions.

Finally, to compare the analyzed papers, the emphasis for the performance metrics was set on accuracy since it describes how the developed solutions perform across all classes (represented by the recognized emotions). Another aspect of accuracy is that it is appropriate to use when all classes are of equal importance, which is pertinent for the emotion’s recognition case.

## 3. Databases Used by FER Systems

An important role in the constant improvements of FER systems is represented by the facial expression databases; this is because collecting an adequate dataset is one of the most critical preliminary aspects for creating automated systems to detect specific classes [25]. Now, the classification rate of emotions is high, but not high enough to obtain a maximum accuracy value. Considering that a person can have a whole spectrum of emotions that can change in a very short time interval, a large training dataset is needed to cover as many cases as possible. Thus, as the required number of detected emotions becomes higher, the more difficult it becomes for the neural networks to distinguish between emotions without having sufficient training data. Additionally, the datasets on which neural networks are trained must be sufficiently diverse because, without diversity, there is a risk for the technology to be biased by minority classification classes [26]. Another aspect is the case of medical conditions or physical impairments where temporary or permanent paralysis of the facial muscles occurs, and the emotions of the concerned persons may be misunderstood by the algorithms [27]. This can lead to a wide range of misclassification situations, with impacts ranging from the receipt of inappropriate services to the misdiagnosis of a psychological disorder. The correct classification rate can also vary from one database to another using the same neural network architecture [28].

Currently, there are a considerable number of databases used for emotion recognition, containing images that vary in size, posture, expressions, lighting conditions, as well as the number of subjects. The images are either acquired in the laboratory or the wild. In the case of images acquired in a controlled environment, the expressions are simulated, and the background has a limited variation, whereas the images acquired in the wild are characterized by a huge variety. Nevertheless, the different environments in which the images were acquired showed that the accuracy of facial emotion recognition results can play an important role in classification based on skin color or ethnicity. It was found that social norms and cultural differences influence the level of expression of some emotions [29].

The field of emotion recognition is emergent, and it needs large databases, obtained especially in the wild where the conditions are very dynamic. The performance of FER systems is highly dependent on the training databases which must be diverse because facial expressions have slight variations from person to person, may mix different emotional states at the same time, or people may not even express emotions.

Table 1 presents the most common databases used in emotion recognition with the aid of neural networks [30]. These databases contain either single images of emotions (of maximum intensity) or sequences of images and videos corresponding to a specific emotion, and other details such as the environment type used for image acquisition, the number of images, the type of images from the color perspective, the number of involved human subjects, and the contained facial expressions that can be observed.

As presented in [29], there are collections of databases that include either

- spontaneous datasets—this refers to expressions that are simulated by the participant. In this case, the participants know the fact that they are monitored, but emotions are shown in a natural way, and in most cases, the acquisition context is a labored one.
- in-the-wild datasets—in this case the process of acquisition is not labored, and the participants are filmed in real-world scenarios.

A problem concerning emotion recognition is represented by micro-expressions [84]. Micro-expressions belong to the domain of non-verbal gestures and can be distinguished by the fact that they refer explicitly to specific situations in which they are likely to appear, as a situation in which the emotion felt is, intentionally or not, hidden. This type of emotion is visible only in a small number of frames, and the facial movement intensity appearing in micro-expressions is very reduced. Therefore, micro-expression recognition requires precise motion tracking and recognition algorithms.

Although micro-expressions are increasingly studied to understand human behavior, they have some characteristics that make their automatic recognition very difficult. These are considered leakages when trying to hide an emotion because they are very short in manifestation time and their truthfulness cannot be measured. Micro-expressions also reveal the true state of a person at a specific time. Such expressions can be easily noticed due to the strong tension of a certain combination of the 55 muscle bundles of the face, which attracts an obvious discrepancy in the series of natural facial expressions of that person [85].

Micro-expressions can also constitute a genuine preamble to certain actions [86]. For instance, they can appear during an interrogation indicating tense areas inside the psyche or they can be visible in stressful situations. Thus, the need for correct identification of facial micro-expressions has led to the creation of databases with images that capture micro-emotions (Table 2). Like facial expressions, the images containing the micro-emotions were acquired either in the wild or spontaneous environment. In the case of micro-expressions from the databases stated above, the expressions are collected quickly, at least in terms of the emotional stimulus presence or absence.

## 4. New Trends in Using Neural Networks for FER

Neural networks are currently used by many artificial intelligence-based applications in domains such as computer vision, machine learning, deep learning, data science, or natural language processing. Neural networks strike a balance between processing time and correct classification rate, and the latest advances have led to the development of complex architectures capable of detecting and classifying patterns by efficiently executing the required operations to determine specific features. In essence, a neural network consists of three important phases:

- Training phase, or backpropagation, in which the network adjusts its parameters to improve its performance by comparing the predictions and ground truth values.
- Validation phase, which is used to compute an unbiased evolution of the generated model against the training dataset.
- Testing phase, or forward propagation, in which the input data are passed through the network components and a final output value (prediction) is given.

Regarding the computer vision domain, neural networks have been successfully used in image classification and more specifically, face identification and facial emotion recognition applications. Besides the main utility in surveillance systems, neural networks have also begun to be used in medical diagnosis applications (to identify patient conditions [69,94,95]) or in applications that involve interaction with a user [96,97,98,99,100].

The specific requirements in the field of face identification and facial emotion recognition have been solved with different types of neural network architectures. For instance, pre-trained networks can be used for the following tasks:

- Classification, which can apply pre-trained networks directly to classification tasks [34,35,38,53,80].
- Feature extraction, which is pre-trained network which can be used as a feature extractor using the activation layers as features, and these layers can be used to train other machine learning models, such as a support vector machine (SVM) [62,77,83,90,101].
- Transfer learning, in which the layers of a neural network trained on one dataset are adjusted and reused to test a new dataset [54,73,102,103,104].

As stated before, DDNs have been increasingly used in emotion recognition due to their promising performances. The following types of DNNs have great popularity, especially in the computer vision field:

- Multi-layer perceptron (MLP)—MLP is the most basic type of DNN; it is composed of a series of fully connected layers, and it can be used to overcome the high computing power requirement of deep learning architectures.
- Convolutional neural network (CNN)—CNN is predominantly used in computer vision to automatically extract features from input data to complete a specific task such as image classification. Features extraction is handled by one or multiple convolutional layers consisting of convolutional operations based on filters, and in this way, CNN models can capture the high-level representation of the input data.
- Recurrent neural network (RNN)—RNN models are suitable for processing sequential data such as time series or text, and they are commonly used in language translation, natural language processing (NLP), speech recognition, and image captioning. Some distinguishing characteristics of RNNs are the parameters sharing across all network layers and the fact that each layer has its own “memory” as information is retrieved from prior inputs and used to influence the current input and output.

Several DNN-based architectures have achieved notable performances in emotion recognition (Table 3).

**Figure 5:**
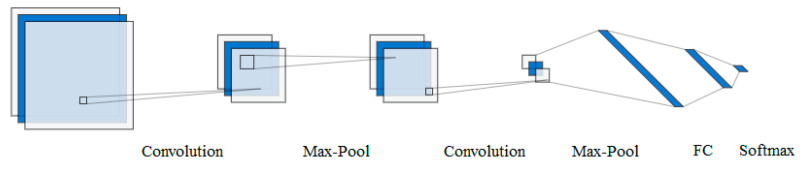
CNN architecture.

The most common DNN-based architecture used in FER systems is represented by a CNN. Figure 5 presents an example of a common architecture used by all CNN models, which consists of a series of convolution and pooling operations, followed by a specific number of fully connected (FC) layers and a SoftMax operation in the case of multiclass classification.

The main properties of the CNN architecture are the local receptive field represented by the process of sharing the neurons’ responsibility for the classification of different parts of an image, weight sharing inside each layer, and spatial subsampling that determines the feature maps size reduction with the preservation of the most important information. Another important aspect of this type of neural network is the absence of the explicit feature extraction step, overcome by the process of implicit learning on the training data which can be processed in parallel, thus reducing the computational cost.

The advantages of choosing a CNN for FER systems include its extremely high level of performance, the elimination of the manual feature extraction requirement since the learning is automatically performed on the training data, and perhaps the most important advantage, which is transfer learning, because CNNs allow subsequent constructions based on initial parts of other pre-trained CNNs [34,71,105,106,107,108,109,110]. Transfer learning can be extremely useful because information learned for one task can be transferred to another task, greatly reducing the processing time by eliminating the need to recollect training data for that given task. Thus, using a pre-trained network with transfer learning is usually much faster than training a network from scratch and it also causes a decrease in the size of the required dataset. *Most of the pre-trained networks are trained on subsets of the ImageNet database* [111]. These networks have trained on more than 1 million images and can classify images into 1000 object categories, such as animals, plants, food, vehicles, etc.

One of the best-known CNN-based neural networks used for different image classification tasks is Google’s Inception network [112]. Being characterized by a rather complex architecture, its constant evolution in terms of speed and accuracy led to the development of a series of versions going from V1 (known also as GoogLeNet) to V4 and, due to ResNet’s performance, a hybrid Inception-ResNet version was even proposed. The base of the Inception networks is represented by the Inception module which consists of a set of convolutional, pooling, and concatenation operations. One particular characteristic of the Inception module is that the convolutional operations use multiple filters of different sizes on the same level, which means that the model becomes wider rather than deeper and the data overfitting issue is avoided. In addition, at the end of the network average, pooling is used instead of fully connected layers, eliminating a huge number of parameters that would not matter. During its architecture evolution on each version, the main goal was to increase the computational efficiency and to decrease the number of parameters, and this optimization gained over each released version was also effective in terms of minimizing the error rate. Therefore, different versions of the Inception network are used for feature extraction in [30] or emotion recognition, transfer learning, and fine-tuning in [62,81,113,114,115,116].

Another architecture with significant performance in emotion recognition is the visual geometry group (VGG) convolutional neural network [117]. The VGG model includes a series of variations including VGG16 or VGG19, which use the same principle but vary only in depth. As the model evolves from simpler to more complex, the network depth increases and a larger number of convolutional layers are put in cascade beside the initial sets of convolutional layers. Although the network size is huge, requiring more time to train its parameters, the VGG architecture has led to promising results, and different VVG variants have been used in many studies so far [32,47,71,102,116,118].

Over the years, the tendency in deep neural networks was to increase the number of layers to reduce the error rate. However, a larger number of layers is a common problem associated with the deep learning field, namely the vanishing/exploding gradient (e.g., the gradient becomes 0 or too large). To overcome this, residual neural network (ResNet) [119] was introduced and its architecture was based on an innovative concept called residual blocks. Essentially, the connection of a layer with further layers is performed by skipping layers in between, which form a residual block. This approach demonstrated that the networks are much easier to optimize, and the accuracy increased proportionally with the network depth. Through different variations of this architectural model, notable results were obtained in the field of emotion recognition [33,39,47,52,84,92,106,120]. Wide ResNet [121], a variant of ResNet, has decreased the depth and increased the width of residual networks. This type of architecture is used in [62] for effective analysis.

AlexNet [122] and LeNet [123] share similar architectures, with the particularity that AlexNet has a much larger number of convolutional layers stacked on top of each other, whereas LeNet has a certain convolutional layer immediately preceded by a pooling layer. In fact, the LeNet pioneering model largely introduced CNNs. The convolutional layers use a subset of the previous layer’s channels for each filter to reduce computation and force a symmetry break in the network, while the subsampling layers use a form of average pooling. It was designed for low-resolution images, and because of time constraints in terms of computing power, it did not present significant results. In [81,124], both networks are used to evaluate the proposed method for facial emotion recognition and in [62,103] for transfer learning.

Further, the Xception architecture [125] abstracts the input of each layer so that in the end it obtains a compact representation of each layer from which a single value is obtained, representing the prediction. The Xception network is used in [54] for feature extraction and in [126,127,128] is used as a data segregator in a pre-trained model.

The YOLOv3 architecture [129] has 53 convolutional layers and aims to replace SoftMax activation mechanisms with independent logistic classifiers. In addition, predictions are made on three distinct scales, which helps the model improve its accuracy in predicting objects. To achieve feature extraction, in [130], the authors use the YOLOv3 face detection model.

EfficientNet [131] is another type of CNN fine-tuned for obtaining high accuracy. This model uses a technique called compound coefficient to scale up models in a simple but effective manner. Instead of randomly scaling up width, depth, or resolution, compound scaling uniformly scales each dimension with a certain fixed set of coefficients. 

NasNet-Large [132] is another convolutional neural network model. Its building blocks consist of normal and reduction cells which return specific feature maps. In case of normal cells, the returned feature maps have the same dimension, whereas reduction cells’ feature maps dimension is reduced by a factor of two. This type of CNN also uses the reinforcement learning search method. In [133], this CNN performed transfer learning for emotion recognition.

The specific CapsNet neural network [134] is used in image processing to try to understand objects in a three-dimensional spectrum. Algorithms such as dynamic routing between capsules can use inverse rendering to decompose objects and to understand the relationships of their views from different three-dimensional angles. Experts highlighted that advances in computing power and data storage have made options such as capsule networks possible. These exciting ideas underlie cutting-edge research into stronger AI. In [135], CapsNet is proposed as the solution for CNNs’ failure to encode different orientation features to recognize facial emotions.

In general, the most used neural network architecture for emotion recognition is the CNN. Whether it is used alone for feature extraction and then for classification, or whether it is used together with another type of network, CNN is without a doubt the type of architecture that has provided the most significant results for both practical applications and for developing theoretical models. In addition, this type of neural network offers the possibility of developing functional solutions in real time (Table 4).

**Figure 6:**
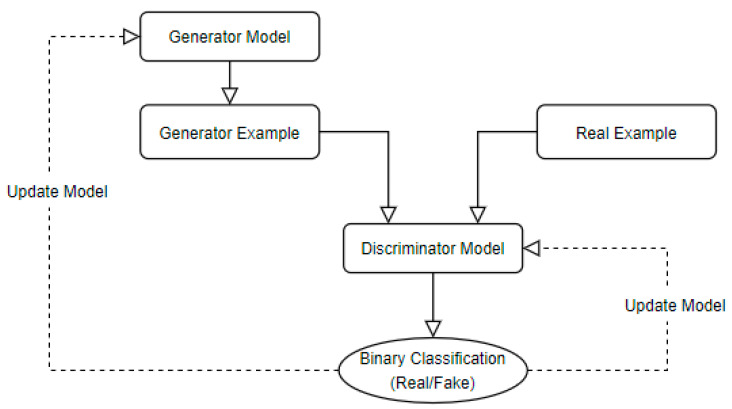
GAN architecture.

Generative adversarial networks (GAN) are also used in FER systems and in the development of any deep neural network that moves towards a higher simulation of human cognitive tasks [80]. Scientists are looking at the potential of generative adversarial networks to increase the power of neural networks and their ability to “think” in a human way because, for instance, in computer vision, GAN is not only trying to reproduce images from training data, but it also trains itself to be able to generate new images, as realistically as possible (Figure 6).

In GAN’s architecture, the network produces outputs from the input and the outputs are passed to a discriminator model, which can distinguish between genuine and synthetic results given by the generative network [143,144]. GAN is also characterized by the flexibility to impose a relational inductive bias in data; in this case, the facial landmarks are seen as a graph to make reasonings about facial attributes and identity [145].

**Figure 7:**
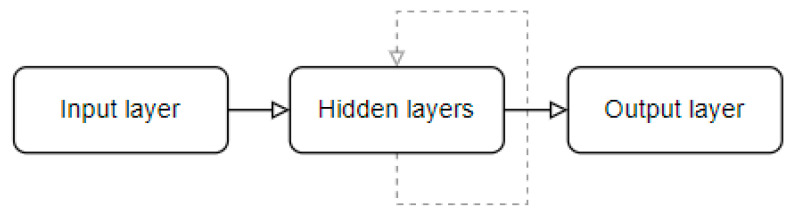
RNN architecture.

Lastly, RNNs are also used in FER systems, particularly long short-term memory (LSTM) RNN architecture, which is specially designed for classifying data that form sequences [146]. The essential difference between networks of this type and classical neural networks is the recurrent layers, where the connections between neurons are cyclic (Figure 7). In the emotion recognition field, RNNs are mostly used for processing image sequences, where each element of the image sequence can depend on the context created by the previous elements of the sequence to recognize emotions. This scenario uses forward propagation and saves data that will be needed in the future. If the prediction is incorrect, the learning rate is used to make minor adjustments. As a result, as backpropagation progresses, it will become more and more accurate [147].

There are also solutions presented in [148,149] where the approach is based on a CNN–RNN mixed model for emotion recognition. Alternatively, one of the latest proposed solutions is to use a specialized neural network called meaningful neural network which learns features from different architectures, algorithms, or descriptive vectors in a “meaningful” way [150]. Another new solution for emotion recognition is the graph neural network (GNN) which opens new possibilities for further research [151].

Although FER systems can detect and recognize human emotions, they are not always 100% accurate because there are many individual variations in terms of expressing and interpreting emotions. Context interpretation is another important aspect of understanding human emotions, and this can be a difficult task to accomplish for artificial intelligence-based systems. 

Nevertheless, the facial emotion recognition process allows the differentiation between friends and enemies, a potential or real threat, being a crucial source of information for social interactions. From this perspective, it is justified to recognize the importance of FER systems. As the level of interpersonal relationships increases, the perception of the interlocutor’s emotions plays an important role in communication between individuals. Furthermore, the automatic recognition of the interlocutor’s emotional state is also important in the context of human–computer interaction, contributing to the gradual removal of some unnatural communication conventions [152].

## 5. Use of the Neural Network-Based FER Systems

In the development of the new methods used in the FER field, an important criterion for comparing emotion recognition solutions from real situations is whether the emotions are spontaneous or simulated. Although research in this field is ongoing, there are existing systems that claim good results from a recognition percentages point of view, but these systems are either still in the initial testing phase using a small number of human subjects, tested on the same dataset that is also used in the training phase or use dramatized emotions (Table 5).

The performances of these methods are on par with the ones described in the literature or even better, but in a real case scenario, these solutions usually achieve low performances.

The technological progress of the FER systems has as a primary purpose of attempting to facilitate the interaction between humans or between humans and the environment. For this reason, the most successful system based on artificial intelligence will be the one that will contain an emotional intelligence as developed as that present in human activities. Implementing such technology will improve the system’s ability to understand emotional input and respond proportionally. This is the reason why domains such as healthcare, education, social IoT, or even standalone systems such as driver assistance systems are integrating FER systems (Table 6).

From Table 7, it can be observed that the solutions developed for practical applications have, in essence, a series of characteristics:

- Multiple used databases.
- Recognized emotions are few and include only basic emotions.
- Tested for real-time use.

Although the interest in the development of practical applications is increasing, most solutions developed for automatic emotions’ recognition are facial emotion recognition solutions developed on a general database which can be then used on a particular dataset (Table 7). In this sense, the researchers have concentrated their efforts on detecting all the main emotions from standardized databases.

The solutions developed for automatic emotion recognition in Table 8 have a series of common characteristics:

- Not tested for real-time use cases.
- Using standardized databases.
- Recognized emotions are the basic ones and variations of them.

Despite recent advances, current models are far from perfect and reliable, and ongoing research is crucial to ensure responsible and ethical use. Assessing content validity is critical and identifying failure modes has become as important as improving performance.

There are also a limited number of papers that use the valence–arousal emotion model which attempts to conceptualize human emotions by defining a scale. In this case, the valence axis indicates how pleasant/unpleasant the emotion is and the arousal axis indicates how high/low the physiological intensity of the emotion is. For these papers, we used the provided concordance correlation coefficient (CCC) as the evaluation criterion for emotion recognition (Table 8), for which a higher value indicates better performance.

## 6. Discussion

### 6.1. Comparison with Similar Review Papers

The existing reviews mainly focus on facial emotion recognition in different scenarios without considering all types of neural networks, and some novel ideas proposed recently are not covered. For example, in [167], the research is focused on different FER techniques in the field of healthcare surveillance systems. Recent papers based on neural networks to recognize emotions are highlighted and inputs such as speech, facial expressions, or audio–visual are used by the neural networks to monitor patients.

In [168] the authors conduct research on CNN-based techniques. This includes an analysis of different CNN architectures with all specific issues for facial emotion recognition and the required steps for using this type of neural network.

The purpose of [169] is to study the recent works on FER solutions via deep learning techniques. The authors presented the architectures of CNN and CNN–LSTM neural networks, the databases used for training and testing, and a summary of the proposed methods along with the obtained results.

In [170], the authors identified the most used methods and algorithms for facial emotion recognition during 2006–2019 for a better understanding together with the FER databases. Neural networks are mentioned as being a classifier in this proposed method, particularly CNNs.

### 6.2. Overview

This paper presents a comprehensive survey of various FER systems based on neural networks. Different challenges and applications of FER systems are also presented in this paper. The main purpose of this paper is to find all the relevant papers from the past five years and to determine the most used neural network architectures based on facial image analyses algorithms for emotion recognition developed on databases consisting of both facial expression and micro-expressions.

With this research, we aim to answer the following questions:

- What neural network architectures based on facial image analysis are predominantly used for emotion recognition?
- What are the major limitations and challenges of FER systems developed with neural networks?

First, this review presents the FER solutions based on neural networks using both facial features and micro-expressions, and for this purpose, a brief presentation of the databases used by FER systems was also made. Further, this review is focused on papers from the last five years (2018–2022) that provide results and because of this, the papers without a clear methodology or without clear experimental results have not been included. This may have excluded some good FER solutions, and studies that have not been peer-reviewed. Similarly, some valuable research may have been excluded prior to the period of the last five years.

Second, an overview of the different types of neural network architectures, especially deep learning models, is presented. A series of classic and advanced CNN, GAN, GNN, and RNN models are analyzed from the perspective of performance obtained in the FER field. Since there are solutions that were trained and tested on the same database, solutions that used different databases, or solutions that were trained and tested on a small number of images, it is difficult to make a comparison between them, especially with the databases that contain either images or sequences. 

Third, advanced deep learning solutions are introduced, especially those that reach state-of-the-art results for facial emotion recognition. Some researchers turn to using different transfer learning techniques to achieve better results. In general, it was concluded in our research that from the neural networks point of view, CNN-based models are currently the leading architectures in FER systems due to their significant results. Nevertheless, other types of architectures such as GNN and RNN promise notable results. Over the past decade, many implementations of FER systems based on different deep learning techniques have shown amazing performance, which in some cases exceeded human performance. For example, in [126], a facial image threshing (FIT) machine for FER datasets is proposed. This solution can transform a dataset used for unsupervised learning to a dataset that can be used for supervised learning by executing tasks such as removing irrelevant images, reorganizing existing sets of images, collecting additional images, or merging images from different datasets. There are also situations in which the proposed methods exceed the state-of-the-art performances [38,39,54,62,74,81,101]. Similarly, context-aware solutions for emotion recognition [47,49,50,98] or practical solutions [37,124,127,130,133,159] demonstrate promising results.

Finally, the applications of FER systems are covered for both real-time and offline use cases. In this sense, the relevant characteristics of the solutions used in different fields such as medicine, IoT, education, and driver assistance, along with the facial emotion recognition procedures, were presented and detailed. In the case of practical and real-time use, it is also observed that there is a growing trend in using a multimodal system to obtain a more accurate FER system. 

Moreover, some of the latest proposals aim to develop FER systems that can be easily extended to dynamic images, abandoning the analysis of static images that are part of a sequence of images and dealing with the problem of detecting and recognizing human emotions in complex scenes from the real world, thus developing appropriate methods for object recognition by respectively extracting the background [166,171]. Another tendency for emotion recognition is the analysis of electroencephalography signals (EEG) with machine learning models. These solutions produce competitive results in terms of accuracy, but the major difficulty is the dataset creation because of the limitations of EEG recorders and human resources [172,173,174].

Although FER systems have recently been improved due to deep learning techniques and technological advances, there are still some limitations to overcome, which include the following:

- Lack of diverse databases causing a need for the acquisition of new large databases with a high level of annotation quality [39,46,53,56,83,124,161,164];
- The proposed methods do not provide better accuracy than the ones described in the literature, or the model achieved performance on par with state-of-the-art methods [49,50,92];
- Misclassifications between emotions (such as ”sad” and “angry”) which indicates that the system needs further improvements [58,120,162,165,175];
- Proposed architectures are usually characterized by high complexity [32,33,41,43,64,78,114,141,163];
- Small number of recognized emotions [45,90,93,116,160];
- The proposed model is built to recognize facial expressions on static images which may limit its applicability [68,73].

FER systems are an emergent field of computer vision research that focuses on developing technologies that can perceive, understand, and respond to human emotions. By integrating with different types of neural networks, the goal is to create artificial intelligence systems that can communicate and interact with people naturally and intuitively, giving them a more human and personalized experience. One possibility could be to integrate the models with vast databases containing information about human emotions and states.

Despite scientific evidence that there is a connection between facial expressions and emotions, the technology is not yet mature enough to accurately trace what the user is feeling. Moreover, facial recognition technology has raised concerns that it could be used to surveil people, which can be translated as a violation of users’ privacy. Analyzing emotions based on facial expressions and body language could be also misleading because these features depend on culture and context. Thus, regulations may need to be put in place to ensure that people continue to be the final decision-makers. 

## 7. Conclusions and Future Work

In this paper, we undertook a review of the new trends in facial emotion recognition using image analysis conducted by neural networks. We also exposed the available datasets that are currently used for emotion recognition from facial expression and micro-expression and the use of different deep learning models in solving this problem. A series of research performed in the FER field were analyzed and the open issues and future trends were addressed.

AI-based systems do not have advanced functions such as perceiving humans’ empathy or understanding human feelings by relating to a context. In the future, we believe that the solutions that will manage to implement a kind of emotional intelligence, through which the creation of typical human reactions will be possible, and in turn these solutions will be more successful. To find an optimized architecture suitable for real-time applications, new techniques are still trying to overcome the difficulties in training, the poor performances, or the computational complexity. However, with the help of embedded boards, various deep learning models can be used with better efficiency. We also believe that the development of real-time multimodal emotion recognition systems will capture the interest of the researchers.

In conclusion, through an automatic emotion recognition system using neural networks, algorithms can analyze facial expressions or micro-expressions that reflect people’s emotions, which are themselves a mirror of their internal state. In this context, emotions are the effect of the presence of a stimulus in the monitored subject, and the interaction is desired to be adapted according to these observations. Although facial emotion recognition has come a long way, the systems are still limited by some technical issues. Nevertheless, because the technology in the FER field is being adjusted continuously in its goals, it holds the potential to revolutionize the science of emotions with the amendment that the algorithms should track people’s movements accurately in their context.

## Figures and Tables

**Table 1:** Facial expressions databases.

Database	Spontaneous/ in-the-wild	Images/ Videos	Type	Subjects	Facial Expression	References
CK+ [31]	spontaneous	593 images	mostly gray	123	neutral, sadness, surprise, happiness, fear, anger, contempt, disgust	[32,33,34,35]
JAFFE [36]	spontaneous	213 images	gray	10	neutral, sadness, surprise, happiness, fear, anger, disgust	[35,37,38,39]
Raf-DB [40]	in-the-wild	8040 images-	color	67	neutral, sadness, contempt, surprise, happiness, fear, anger, disgust	[41,42,43]
AffectNET [44]	in-the-wild	~450,000 manually ~500,000 automatically annotated	color		neutral, happiness, sadness, surprise, fear, disgust, anger, and contempt	[33,45,46,47]
Aff-Wild2 [48]	in-the-wild	~2,800,000 manually annotated	color	458	neutral, happiness, sadness, surprise, fear, disgust, anger + valence–arousal + action units 1,2,4,6,12,15,20,25	[49,50]
FER-2013 [51]	in-the-wild	35,000 images	gray		angry, disgust, fear, happiness, sadness, surprise, neutral	[52,53,54]
ADFES-BIV [55]	spontaneous	370 videos		12	anger, disgust, fear, joy, sadness, surprise, contempt, pride, embarrassment	[56]
WSEFEP [57]	spontaneous	210 images	color	30	enjoyment, fear, disgust, anger, sadness, surprise, neutral	[56,58]
OAHEGA [59]	in-the-wild	15,744 images	color		neutral, happy, angry, surprise, sadness	[52]
KDEF [60]	spontaneous	490 images	grey	272	angry, fearful, disgust, happiness, sadness, surprised, neutral	[37,61,62]
Oulu-CASIA [63]	spontaneous	480 sequences	color	80	surprise, happiness, sadness, anger, fear, disgust	[64,65]
SASE-FE [66]	spontaneous	600 videos	color	50	anger, happiness, sadness, disgust, contempt, surprise	[34]
SFEW [67]	in-the-wild	1739 images	color	330	anger, disgust, fear, neutral, happiness, sadness, surprise	[68,69]
AFEW [70]	in-the-wild	1426 sequences	color	330	anger, disgust, fear, happiness, sadness, surprise, neutral	[65,71,72,73,74]
iCV-MEFED [75]	spontaneous	31,250 images	color	125	anger, contempt, disgust, fear, happiness, sadness, surprise, neutral	[30]
MMI [76]	spontaneous	2900 videos	color	75	sadness, happiness, fear, anger, surprise, and disgust	[71,73,74,77,78]
Multi-PIE [79]	spontaneous	750,000 images	color	337	neutral, smile, surprise, squint, disgust, scream	[80,81]
IEMOCAP [82]	spontaneous	12 h video	color	120	anger, happiness, excitement, sadness, frustration, fear, surprise, neutral	[83]

**Table 2:** Micro-expression facial datasets.

Database	Characteristics	Images	Subjects	Facial Expression	References
SMIC [87]	spontaneous	164	6	77 micro-expressions	[64,84,88,89,90]
CASME II [91]	spontaneous	247	26	happiness, disgust, surprise, repression, and others	[64,84,88,89,90,92,93]
SAMM [94]	spontaneous	159	32	contempt, disgust, fear, anger, sadness, happiness, surprise	[64,89,90,92]

**Table 3:** DNN-based architectures used by FER systems.

Architecture	Type
CNN	ResNet12, ResNet18, ResNet34, ResNet50, ResNet56, ResNet92, ResNet101, 2D-ResNet, ResNetXt34, SE-ResNet34, SE-ResNeXt34, SE-ResNet50, EmoResNet, VGG11, VGG14, VGG16, VGG17, VGG19, VGG-M, InceptionV3, InceptionV4, InceptionResNetV2, Xception, Mini_Xception, GoogleNet, GoogleLeNetv2, LeNet, YOLOv3, EfficientNet, AlexNet, NasNet-Large, Wide ResNet, LEMHI-CNN, CNN –RNN, CAER-Net, CAER-Net-S, ArcFace CapsNet with No FL, FL-CapsNet, MTCNN
GAN	GAN, 2k GAN
GNN	GNN
RNN	LSTM, EmoNet

**Table 4:** CNN architecture used for FER systems.

Reference	Architecture	CNN Used	Emotions Detected	Accuracy	Proposed Solution Description	Limitation of the Proposed Solution
[101]	CNN SVM	feature extraction/ classification	7	99.69%(CK+), 94.69% (BU4D)	A new framework for facial expression recognition by using a hybrid model.	Developed only on western databases for the recognition of facial expression.
[81]	AlexNet, GoogLeNet, LeNet	feature extraction/ classification	8	99.93% (Multi-PIE), 98.58% (CK+)	Multiple CNNs using improved fuzzy integral were proposed for recognition facial emotions.	Need to eliminate lower or similar classifiers to achieve the best combination of classifiers.
[120]	ResNet50	feature extraction	9	85% (Caltech-256)	An efficient scheme for inferring emotion tag from object images.	Suffers from the problem of subjectivity.
[136]	Two-level CNN	feature extraction/ classification	5	45% (CK+), 85% (Caltech faces), 78% (CMU), 96% (NIST), All datasets: 96%	A novel technique called facial emotion recognition using CNN.	The algorithm failed when multiple faces were present in the same image, at an equal distance from the camera.
[74]	LBP, 3D CNN	feature extraction/ classification	7	96.23% (CK+), 96.69% (MMI), 99.79% (GRMEP-FERA), 31.02% (AFEW)	A robust multi-depth network that can efficiently classify facial expressions through feeding various and reinforced features.	For the CK+ database, the proposed scheme did not obtain the best result compared with some existing models.
[137]	Viola–Jones algorithm, Haar-like feature, CNN	classification	7	94.94% (cross dataset JAFFE, CK+), 92.66% (mixed datasets)	New architecture design for a CNN for the FER system.	Not using dark-colored faces and dark images for emotion recognition.
[138]	ResNet101 Faster R-CNN	feature extraction/ classification	8	75.46% (F1), 84.71% (IAPsubset), 74.58% (ArtPhoto), 70.77% (Abstract), 82.84% (EmotionROI)	A framework to automatically detect emotional regions on multi-level deep feature maps.	The relationship between different emotions can be exploited to predict emotion distribution more precisely.
[139]	Haar cascade CNN	feature extraction/ classification	7	88.10% (FER13)	A hybrid CNN to recognize human emotions into sub-categories.	Lack of diverse databases.
[103]	AlexNet	feature extraction/ classification	7	99.44% (CK+), 70.52% (FER2013)	A deep learning method based on transfer learning.	The model’s accuracy trained on the augmented CK+ dataset dropped by 3%.
[140]	MT-CNN, Light-CNN, dual-branch CNN, pre-trained CNN	feature extraction/ classification	8	95.29% (CK+), 86.50% (BU-3DEF), 71.14% (FER2013)	Three CNN models for facial expression recognition in the wild.	Need efficient hand-crafted features.
[135]	Viola–Jones algorithm, FL-CapsNet	classification	8	98.27% (JAFFE), 8.82% (CK+), 77.99% (FER2013)	A face localization algorithm for emotion recognition.	The learning rate has impacted the model training and affected the recognition accuracies.
[104]	Transfer learning VGG16 and ResNet50 PCA	feature extraction/ classification	6	76.2% (FER-2013), 99.4% (CK+), 99.6% (FERG-DB), 88.68% (combined)	A precision-based weighted blending distributed ensemble model for emotion classification.	Poorest performance when classifying the ”disgust” and ”surprise” emotions.
[62]	VGG16, ResNet50, Inception ResNet, Wide ResNet, AlexNet, Correlation analysis SVM	feature extraction	6	99.22% (JAFFE), 99.78% (CK+), 92.78% (FER 2013), 96.32% (KDEF)	A novel transfer learning-based FE feature extraction approach using DNN and correlation analysis.	The methodology proposed to obtain significant results only uses the databases obtained in a controlled environment.
[42]	VGG-11, VGG-16, ResNet50, 2D CNN–LSTM, I3D-CNN	feature extraction/ classification	7	79.9% (RAF-DB)	Two CNN architectures for continuous emotion prediction in the wild.	Use of Aff-Wild dataset to exploit occlusion cases, pose variations, or even scene breaks.
[78]	AlexNet, VGG11, 2k GAN	feature extraction/ classification	7	59.62%(JAFFE), 76.58% (CK+), 61.86%(MMI)	An unsupervised domain adaptation method to improve the cross-dataset performance of facial expression recognition.	Network complexities.
[133]	Fast R-CNN, NasNet-Large CNN	feature extraction/ classification	8	99.95% (FER2013), 98.48% (JAFFE), 99.73% (CK+), 95.28% (AffectNet), 99.15% (Custom dataset)	An algorithm for recognizing the emotional state of a driver.	Network complexities.
[37]	DenseNet-161	feature extraction/ classification	7	96.51% (KDEF), 98.78% (JAFFE)	Efficient DCNN using TL with pipeline tuning strategy for emotion recognition from facial images.	Datasets with low-resolution images or with highly imbalanced cases will need additional preprocessing and appropriate modification in the method.
[92]	ResNet18, ImageNet	feature extraction/ classification	5	60.17% (CASME II, SAMM)	Cost-efficient CNN architectures to recognize spontaneous micro-expression	The method does not provide better accuracy than the ones described in the literature.
[32]	VGG16, ResNet50 with MLP	feature extraction/ classification	7	100% (CK+), 96.40% JAFFE), 98.78%(KDEF)	Facial emotion recognition procedure.	Network complexities.
[33]	ResNet18, ViT-B/16/S, ViT-B/16/SG, ViT-B/16/SAM	feature extraction/ classification	7	50.05% (FER2013, CK+, AffectNet)	Fine-tuned ViT with a FER-based base configuration for image recognition.	Network complexities.
[116]	VGG-16, GoogleNet	feature extraction/ classification	3	71.91% (EMOd, CAT2000)	The improved metric for evaluating human attention that takes into account human consensus and image context.	A small number of emotions recognized.
[39]	2D-ResNet	feature extraction/ classification	6	99.48% (JAFFE)	Easily identifies maskable and skeptical-covered image expressions at a high hit rate.	Lack of diverse databases.
[45]	InceptionResNetV2	feature extraction/ classification	4	79.5% (AffectNET)	Consolidated results for the approach of mouth-based emotion recognition	A small number of emotions recognized.
[84]	ResNet-56, ResNet-92, EmoResNet	feature extraction/ classification	6	91% (CASME II, USF-HD, SMIC)	Detects the actual expressions at the micro-scale features.	The input images must be taken with at least 1 200fps camera and high-resolution quality images are needed.
[141]	A binary CNN (B-CNN) and an eight-class CNN (E-CNN)	feature extraction/ classification	8	64.6% (Image Emotion Dataset, IASP-subset, ArtPhoto, Abstract paintings)	A novel CNN and an assisted learning strategy for emotion recognition.	Network complexities.
[71]	LEMHI-CNN CNN–RNN, VGG	feature extraction/ classification	7	78.4% (MMI), 3.9% (CK+), 51.2% (AFEW)	Facial expression recognition framework.	To improve the performance, the architecture proposed needs to be further explored.
[142]	CNN	feature extraction/ classification	7	95.65% (JAFFE), 99.36% (CK+)	An efficient deep learning technique for classifying emotions.	Lack of diverse databases.
[126]	MTCNN, Xception	feature extraction/ classification	8	60.99% (FER 2013), 86.66% (CK+), 99.22% (iSPL)	A facial image thresholding machine for the facial emotion recognition dataset manager.	The model failed to generalize the outside world’s facial emotions.
[106]	ResNet18, ResNet12	feature extraction/ classification	8	99.31% (CK+), 84.29% (FER+)		

**Table 5:** FER solutions tested on a small number of human subjects and on the same database.

Reference	Method	Database	Accuracy
[37]	DenseNet-161	KDEF/4900 images JAFFE/213 images	96.51% 98.78%
[38]	CNN	CK+/593 images JAFFE/213 images	97.05% 98.63%
[88]	CNN	CASME II/247 images SMIC/164 images	69.92% 54.84%
[61]	VGG16	KDEF/4.900 images	88%
[142]	CNN	JAFFE/213 images CK+/3150 images	95.65% 99.36%
[102]	VGG19	CK+/593 images JAFFE/213 images	96.46% 91.27%
[72]	ResNet18	CK+/593 video sequences AFEW 8.0/1.809 samples	99.69% 51.18%
[89]	VGG-M, OC-NET	SMIC/164 images CASME II/145 images SAMM/132 images	74.8% 90.8% 71.72%
[77]	GoogleLeNetv2	CK+/593 sequences MMI/5130 images RaFD/67 images	98.38% 99.59% 99.17%
[153]	ResNet101	KDEF/4.900 images JAFFE/213 images RaFD/8.040 images	94.59% 92.86% 98.88%
[154]	CNN	CK+/327 images JAFFE/213 images	93.46% 94.75%
[155]	RNN	CK+/327 images	95.4%
[65]	ResNet50	CK+/593 images Oulu-CASIA/80 images AFEW/1809 images	98.46% 87.31% 53.44%

**Table 6:** Relevant FER solutions across different areas.

Field of Use	Reference	Year	Accuracy per Data Source	Emotion Detected	Real-Time
medicine	[156]	2019	93%—dataset collected	3	yes
	[118]	2020	69.25%—BVDB, 64.35%—SEDB	1	no
	[69]	2021	82.63%—KDEF, 96.75%—GENKI, 96.81%—CK+, 36.79%—SFEW	7	no
	[93]	2021	96.2%—dataset of emotions recorded in laboratory (69 patients)	1	no
	[45]	2020	79.5%—AffectNET	4	no
	[35]	2022	87.05%—FER13, 99%—JAFFE, 98.79%—CK+	6	yes
	[127]	2022	87.5%—FER13	7	yes
	[130]	2022	89.31%—LIRIS, 90.98%—author’s dataset	7	no
social IoT	[126]	2021	60.99%—FER13, 86.66%—CK+, 99.22%—iSPL	8	no
	[157]	2022	74.14%—FER2013 and self-collected dataset	7	yes
	[158]	2021	FER2013—69%	6	yes
	[113]	2020	90.14%—ResNet/FER2013, 87%—VGG/FER2013, 81%—Inception V3/FER2013	7	no
	[58]	2020	57.28%—database collected	7	no
	[128]	2021	73%—custom database	3	yes
	[116]	2019	71.91%—EMOd, CAT2000	3	no
	[34]	2022	84.58%—mixed	8	yes
	[84]	2021	91%—custom database	6	yes
	[97]	2022	67.7%—HELEN	5	no
	[98]	2022	99.48%—images, 89.78%—videos experiment1, 90.84%—videos experiment2	6	yes
	[124]	2019	93.03%—custom database	8	yes
driver assistance system	[54]	2022	99.31%—FER-2013, 99.29%—CK+	7	no
	[47]	2021	89%—AffectNET and database collected	8	no
	[159]	2022	84.41%—FER 2013, 95.1%—CK+, 98.50%—KDEF, 98.60%—KMU-FED	7	yes
	[115]	2022	96.6%—FER-2013, CK+, data collected	7	yes
	[133]	2022	99.95%—FER2013, 98.48%—JAFFE, 99.73%—CK+, 95.28%—AffectNet, 99.15%—custom dataset	8	no

**Table 7:** Relevant FER solutions built on standard databases.

Reference	Accuracy per Database	Emotion Detected	Real-Time Use Cases	Reference	Accuracy per Database	Emotion Detected	Real-Time Use Cases
[56]	95.12%—WSEFEP	10	no	[30]	51.84%—dataset collected iCV-MEFED	50	no
[37]	96.51%—KDEF, 98.78%—JAFFE	7	no	[160]	84.68%—GroupEmoW	3	no
[92]	60.17%—CASME II, SAMM		yes	[141]	64.6%—image emotion dataset, IASP-subset, ArtPhoto, sbstract paintings	8	no
[38]	97.05%—CK+, 98.63%—JAFFE	7	no	[71]	78.4%—MMI, 93.9%—CK+, 51.2%—AFEW	7	no
[32]	100%—CK+, 96.4%—JAFFE, 98.78%—KDEF	7	no	[161]	93.24%—CK+, 95.23%—JAFFE	7	no
[53]	58%—FER2013	7	yes	[96]	98.65%—JAFFE, 70.14%—FERC-2013	7	no
[33]	50.05%—FER2013, CK+48, AffectNet	7	no	[142]	95.65%—JAFFE, 99.36%—CK+	7	no
[88]	69.92%—CASME II, 54.84%—SMIC	3	no	[102]	96.46%—CK+, 91.27%—JAFFE	6	no
[39]	99.48%—JAFFE	6	no	[41]	85.59%—RAF-DB, 67.96%—FER2013	7	no
[68]	90.48%—CK+, 89.01%—JAFFE, 50.12%—SFEW	6	no	[126]	60.99%—FER 2013, 86.66%—CK+, 99.22%—iSPL	8	no
[78]	59.62%—JAFFE, 76.58%—CK+, 61.86%—MMI	7	no	[101]	99.69%—CK+, 94.69%—BU4D	7	no
[46]	59%—AffectNet	8	no	[72]	99.69%—CK+, 51.18%—AFEW	8	no
[80]	87.08%—Multi-PIE, 73.13%—BU-3DEF	6	no	[162]	70.02%—FER2013, 98%—CK+, 92.8%—JAFFE, 99.3%—FERG	7	no
[163]	77.04%—CAER, 73.51%—CAER-S	6	no	[89]	74.8%—SMIC, 90.8%—CASME II, 71.72%—SAMM, 79.14%—overall	3	no
[106]	99.31%—CK+, 84.29%—FER+	8	no	[73]	98.47%—CK+, 69.64%—MMI, 50.65%—AFEW	7	no
[81]	99.93%—Multi-PIE, 98.58%—CK+	8	no	[74]	96.23%—CK+, 96.69%—MMI, 99.79%—GRMEP-FERA, 31.02%—AFEW	7	no
[22]	71.13%—eNTERFACE’05, 65.9%—RAVDESS, 52.14%—CMEW	6	no	[140]	95.29%—CK+, 86.5%—BU-3DEF, 71.14%—FER2013	8	no
[136]	45%—CK+, 85%—Caltech faces, 78% -CMU, 96%—NIST, 96%—all datasets used	5	no	[104]	76.2%—FER-2013, 99.4%—CK+, 99.6%—FERG-DB, 88.68%—combined	6	no
[137]	94.94%—cross dataset JAFFE, CK+, 92.66%—mixed datasets JAFFE, CK+	7	no	[62]	99.22%—JAFFE, 99.78%—CK+, 92.78%—FER 2013, 96.32%—KDEF	6	no
[73]	60.7%—AffectNet	8	no	[77]	98.38%—CK+, 99.59%—MMI, 99.17%—RaFD	6	no
[138]	75.46%—F1, 84.71%—IAPSsubset, 74.58%—ArtPhoto, 70.77%—abstract, 82.84%—EmotionROI	8	no	[90]	56.5%—CASME II, 43.7%—SMIC, 36.9%—SAMM, 88.2%—combined	3	no
[139]	88.1%—FER13	7	no	[42]	79.9%—RAF-DB	7	no
[103]	99.44%—CK+, 70.52%—FER2013	7	no	[83]	71.04%—IEMOCAP	4	no
[164]	91.89%—FER2013	6	no	[114]	99.66%—JAFFE, 90.16%—FER2013	7	no
[153]	94.59%—KDEF, 92.86%—JAFFE, 98.88%—RaFD	8	no	[154]	93.46%—CK+, 94.75%—JAFFE	6	no
[50]	66.8%—Aff-Wild2	7	no				

**Table 8:** Relevant papers performance using the valence–arousal model.

Ref.	Architecture	Valence CCC per Database	Arousal CCC per Database
[143]	CNN	0.791—AVEC2016	0.805—AVEC2016
[15]	RNN	0.676—RECOLA	0.446—RECOLA
[148]	CNN, RNN	0.535—Aff-Wild, Aff-Wild2	0.365—Aff-Wild, Aff-Wild2
[46]	CNN	0.71—AffectNet, 0.75—SEWA, 0.57—AFEW-VA	0.63—AffectNet, 0.52—SEWA, 0.56—AFEW-VA
[165]	LSTM	0.068—LIRIS-ACCEDE	0.128—LIRIS-ACCEDE
[47]	CNN	0.408—AffectNet	0.373—AffectNet
[166]	ANN	0.75—SEWA, 0.438—Aff-Wild2	0.64—SEWA, 0.498—Aff-Wild2
[42]	2D CNN–LSTM	0.625—RAF-DB	0.557—RAF-DB
[50]	CNN–RNN	0.505—Aff-Wild2	0.475—Aff-Wild2

## Data Availability

Not applicable.

## Abbreviations

AI	Artificial intelligence
ANN	Artificial neural networks
AU	Action units
CNN	Convolutional neural network
CCC	Concordance correlation coefficient
DNN	Deep neural network
EEG	Electroencephalogram
FC	Fully connected
FER	Facial emotion recognition
GAN	Generative adversarial networks
GNN	Graph neural network
HOG	Histogram of oriented gradients
IoT	Internet of things
KNN	K-nearest neighbors
LFA	Local feature analysis
LDA	Linear discriminant analysis
LBP	Local binary pattern
LSTM	Long short-term memory
MLP	Multi-layer perceptron
NLP	Natural language processing
PRISMA-ScR	Systematic Reviews and Meta-Analyses extension for Scoping Reviews
PCA	Principal component analysis
RNN	Recurrent neural network
ResNet	Residual neural network
SVM	Support vector machine
VGG	Visual geometry group
